# Into the Unknown: How Computation Can Help Explore Uncharted Material Space

**DOI:** 10.1021/jacs.2c06833

**Published:** 2022-10-07

**Authors:** Austin M. Mroz, Victor Posligua, Andrew Tarzia, Emma H. Wolpert, Kim E. Jelfs

**Affiliations:** † Department of Chemistry, Molecular Sciences Research Hub, Imperial College London, White City Campus, Wood Lane, London, W12 0BZ, U.K.

## Abstract

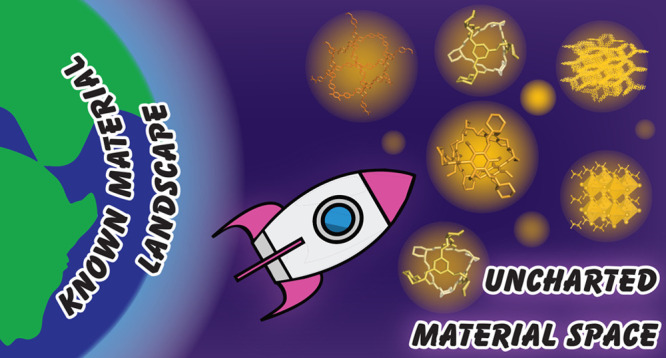

Novel functional materials are urgently needed to help combat the major global challenges facing humanity, such as climate change and resource scarcity. Yet, the traditional experimental materials discovery process is slow and the material space at our disposal is too vast to effectively explore using intuition-guided experimentation alone. Most experimental materials discovery programs necessarily focus on exploring the local space of known materials, so we are not fully exploiting the enormous potential material space, where more novel materials with unique properties may exist. Computation, facilitated by improvements in open-source software and databases, as well as computer hardware has the potential to significantly accelerate the rational development of materials, but all too often is only used to postrationalize experimental observations. Thus, the true predictive power of computation, where theory leads experimentation, is not fully utilized. Here, we discuss the challenges to successful implementation of computation-driven materials discovery workflows, and then focus on the progress of the field, with a particular emphasis on the challenges to reaching novel materials.

## Introduction

The discovery of new materials has the power to transform our lives. For example, new battery materials have allowed miniaturized devices with increased power that have revolutionized the electronics industry. Advanced functional materials are necessary to address the global challenges humanity faces, such as resource scarcity with continued population growth, along with climate change. These challenges include the drive toward clean energy, targeted medical therapies, and assistive technologies for improved quality of life. New materials, including those with improved properties through novel structures, are needed to help us meet ambitious targets put forward by the EU Green Deal^1^ and US Department of Energy.^2^

Ideally, we would have the ability to specify a set of properties required in a multifunctional material and then be able to directly design and realize that material; this is known as inverse design. For every task, a material needs to be tailored to meet the necessary criteria, including target properties and, importantly, the cost and ease of processing into the necessary device form. To take one example from molecular separations, it is estimated that molecular separations account for 10–15% of the world’s energy usage.^3^ Most separations are currently performed using energy intensive distillation processes; however, if these separations could be replaced with a membrane-based process, they could use 90% less energy.^3^ Important molecular separations include separating different hydrocarbons from crude oil, uranium from seawater, greenhouse gases from dilute emissions, rare-earth metals from ores, and trace contaminants from water. Each of these tasks will need a different membrane material, perfectly tailored to a given operation, and new ones will need to be developed for renewable-based separations as we move away from an oil-based economy.

**Figure 1:**
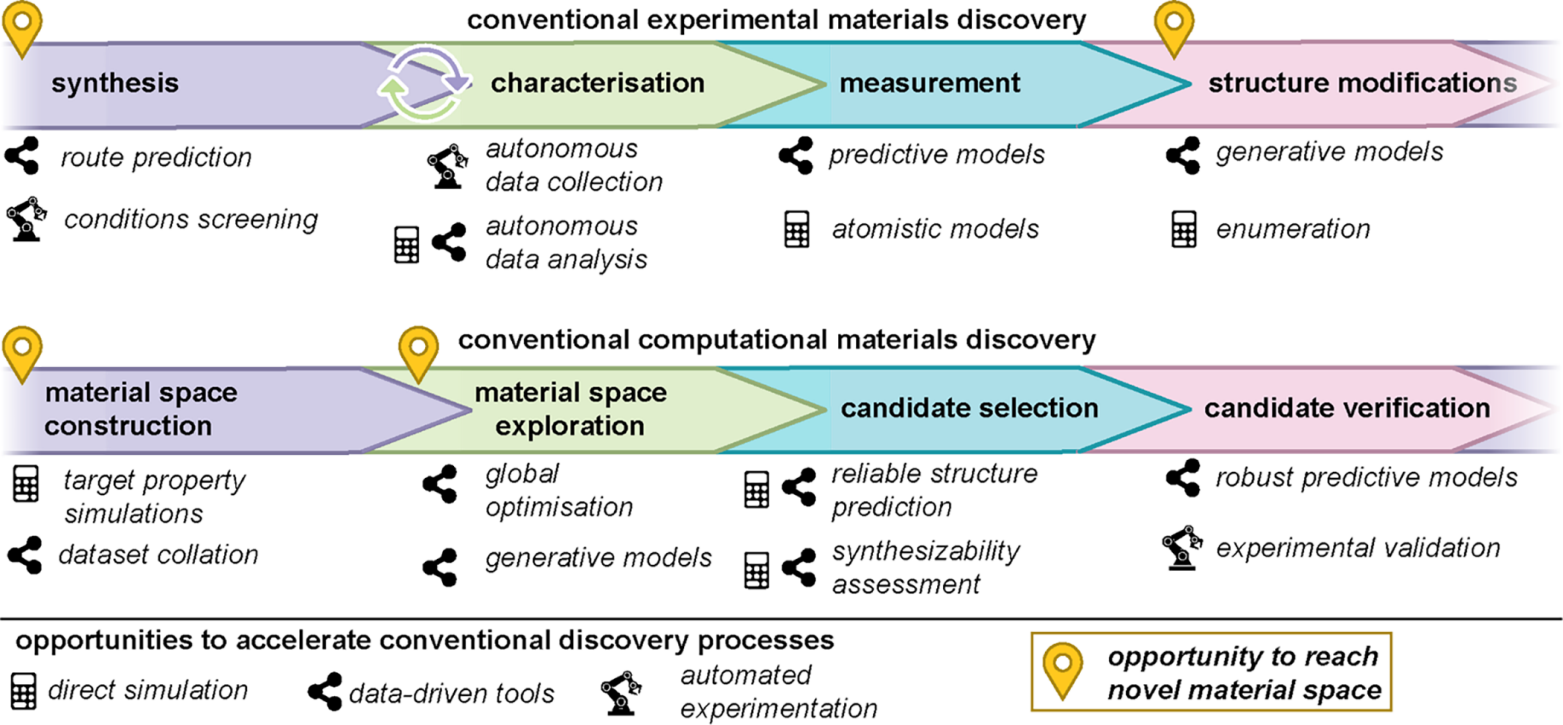
Conventional materials discovery workflows. The typical steps in conventional experimental and computational materials discovery workflows. Specific opportunities to accelerate the conventional discovery process are highlighted, as well as steps in the discovery process possessing a higher likelihood of exploring novel material space.

Traditional materials discovery approaches, Figure 1, typically have long timeframes (up to 20 years on average).^4,5^ Generally, discovery is guided by the scientist’s experience and knowledge of a given material class that will suggest, for example, the addition of a different functional group or heteroatom, or the substitution of a metal or cation to an existing material with the goal of improved performance. The synthesis and characterization of each individual material may take months to years, and thus a knowledge-guided “trial-and-error” process is inherently slow. Worse, given not all syntheses will be successful, and a synthesized material may not produce the desired properties, there is a natural inclination toward only small iterations to known materials due to the risk that larger steps away from known materials are completely unsuccessful, wasting time and resources.^6^ Thus, large leaps forward, for example the discovery of a completely novel material class with previously unrealized properties, are rare and generally a product of serendipity rather than design.

The restrictions on our ability to explore new material space are particularly stark when placed in the context of the size of the potentially available material space. Considering just drug-like (small) organic molecules as potential components of materials, it has been estimated that there are between 10^23^ and 10^60^ hypothetical molecules that could be enumerated through different connectivities and substitutions.^7^ In contrast, it is believed that only a small fraction of these molecules have ever been synthesized by humans, and of course, very few of these have been formed into materials and tested for their potential properties. Moreover, the number of hypothetical molecules highlighted here only describes the phase space spanned by purely organic molecules and the introduction of inorganic elements and materials, for which both the possible elemental compositions and stoichiometries can be tuned, leading to a near infinite phase space. This already enormous material space generated by the combinations of constituent building blocks is further expanded when variety in composition and structure is considered; from discrete or continuous topologies that produce crystalline or amorphous phases to a vast number of forms including thin films, membranes, fibers, and multicomponent devices.

Computation has played an increasing role in materials development over the past decades, aided by substantially increased computer power. The traditional role of computation in materials discovery has been to postrationalize experimental observations—greatly impacting our understanding of the atomistic origin of material properties and structure-property relationships. While this understanding has naturally fed into rational development of improved materials in the laboratory, ideally computation would be used in a truly predictive manner to guide synthesis and allow us to access novel material architectures with optimal properties. With computation, we do not need to be limited to local material space due to the risks of failed syntheses, and there is the potential to computationally screen thousands to millions of hypothetical materials in the time frame that a single material can be tested in the laboratory. Thus, predictive computational capabilities can open the potential to fully exploit the vast potential material search space.

The past decade has seen an increase in the use of data-driven techniques, with the application of artificial intelligence (AI), in particular, machine learning (ML). The increased use of ML in materials development has been facilitated by open science, with open-source algorithms, databases, and improved computer hardware all playing a role. The use of ML in understanding and designing materials and best practices have been reviewed elsewhere.^8−15^ Owing to its ability to identify subtle trends in high-dimensional data, ML can play a variety of roles in materials discovery, from accelerating property prediction by multiple orders of magnitude, to the generation of novel material structures that might not be suggested by scientists alone, to automated extraction of literature data and the optimization of syntheses or properties. However, to date, there are still limited examples of experimental realization of predictions resulting from ML. Thus, it could be argued that presently we do not use computation to its full potential. Effective and robust computation-guided synthesis would allow exploration of an enormous search space, and limit the need for time-consuming experimental testing. Indeed, moving away from traditional materials development approaches (Figure 1) is fueled by strengthening ties between experiment and computation.

Current endeavors linking experiment and computation are being fueled by advances in joint workflows, high-throughput robotics, experimental automation, and AI. Yet, we must ensure that the advances here translate to accelerated discovery of novel architectures to realize their full potential. In this perspective, we discuss the barriers to computational discovery of *novel* material space, followed by a discussion of the progress toward overcoming these barriers that will allow us to achieve true inverse design of novel architectures via computation, highlighting several recent examples. Here, we make the distinction between the three major avenues encompassed by materials development, (i) *identification*, where materials are selected from defined data sets, (ii) *design*, where small modifications are made to known chemical architectures, and (iii) *discovery*, where truly novel architectures are accessed. Conventional materials development studies largely follow either an identification or design approach (or a combination of the two). Owing to the diverse topological landscape, as well as complex synthetic routes, materials *discovery* is challenging with the current tools available.

## Challenges for Computation-Led Exploration of Novel Material Space

Before discussing the challenges to the exploration of novel material space, it is useful to explore how typical computational workflows might be used to assist the materials discovery process, Figure 1. A workflow typically follows several stages: (i) problem definition, (ii) determination of the chemical/material space to be explored, (iii) structure prediction, (iv) exploration of the chemical space with property prediction, and (v) identification of promising target materials for synthesis.

Within problem definition, the target property and the materials class of interest are defined. The materials class is often defined as a specific type, although we might imagine an ideal future where this is kept as open and broad as possible. Consider, for example, metal–organic frameworks (MOFs); the size of the material space for MOFs is, in theory, enormous, since any metal complex may be combined with any organic linker within chemical constraints. In practice, the material space might be defined by selecting a library of precursors to be enumerated—here, a severe restriction in chemical space is often introduced, with small libraries on the order of 10s of precursors selected, since the enumeration still leads to a larger number of hypothetical materials. Alternatively, different material space exploration approaches can be used where the material components can be modified on-the-fly, for example through a set of predefined rules. Ideally, one would want to be able to modify the materials’ structures continuously to allow optimization of desired properties.

The structure of the material must next be predicted. This may require a solid-state, three-dimensional prediction of structure, or, for some data-driven approaches, only the underlying connectivity of the structure. Thereafter, the properties of the materials can be calculated from the structural description. A wide variety of molecular simulation approaches can be used for both structure and property prediction, from coarse-grained or atomistic classical mechanics to a quantum mechanical description, featuring a wide range of associated computational costs. Alternatively, if a sufficient quantity and quality of data are available, ML can be used. Naturally, the accuracy and computational cost strongly influence the size of the material space that can be explored, ranging from 10s to millions of hypothetical materials. The size of the material space will also guide the choice of exploration approach used, from a brute-force combinatorial approach where all possibilities (within the defined space) are tested, to the use of methods such as evolutionary algorithms and Bayesian optimization to efficiently locate promising materials.

### Structure Prediction

The accurate prediction of the molecular and/or solid-state three-dimensional structure of a hypothetical material is essential to accurately predicting material properties and device performance. The significant challenge that solid-state structure prediction poses, for either inorganic or organic solids, has been widely discussed,^16−18^ and the difficulty of this prediction is arguably one of the largest challenges to human or computer-led design of any chemical system. The solid-state arrangement of a system is a key driver in that system’s properties. Thus, without the ability to reliably predict solid-state structure, it is not possible to screen that material. For example, the solid-state packing of molecules can impact the diffusion of guest compounds, influencing their application in sensing. Even small changes to a system, e.g. switching halogens, can change the solid-state arrangement and, thus, system properties.

Increasingly, global optimization searches of possible assemblies have demonstrated the capability of computation to predict the solid-state structure of materials. Through this, accurate energetic assessments are conducted with the expectation that the lowest energy structures should be experimentally observed.^19,20^ However, the challenge in computational structure prediction is that accuracy is closely tied with computational cost, often requiring electronic structure-based methods for accurate energy assessment of possible assemblies. Therefore, at least in the short term, it is not possible to apply these methods to large numbers of hypothetical materials and thus difficult to branch far into truly novel material space.

### The Challenge of Complexity

While the computational approaches developed over the years have provided invaluable atomistic insight into the origin of observed properties beyond those available from experiment alone, model simplifications are necessary to accommodate resource availability. The quantitative accuracy of a computational model stems from its suitability in describing the system. There are many well-established reasons why computational models incur errors.^21−23^ For example, errors can arise from size consistency or size extensivity problems that are intrinsic to the method—larger systems sometimes embody significant medium- and long-range interactions (e.g., van der Waals forces, electrostatic, dipolar, and Coulomb interactions) or self-interaction error that might not be noticeable in small test cases. While model abstractions improve computational efficiency, these simplifications often mean we neglect the true complexity of the material, thereby limiting our ability to fully predict the properties. More computationally affordable methods are often empirically or semiempirically derived, and, thus, can lack transferability to new systems. The latter is a particular issue if the goal of the simulations is to explore truly novel material space.

It is important to ensure that the assessment of a potential material truly considers all the factors that influence device level performance. Often, optimization of device performance and material properties are completed as two separate tasks, necessitated by the complexity of this multivariable optimization problem. We refer the reader to reviews of device-level optimization for solar cells,^24^ field-effect transistors,^25^ thermoelectrics,^26^ and batteries.^27^ While screening bulk “idealized” materials may be useful to remove materials without suitable properties, screening only bulk material properties is unlikely to identify the best materials at a device level. Factors that can influence device level performance include (i) device assembly, which often includes a combination of different materials, (ii) interface between these materials, which can have a disproportionate influence on performance, (iii) operating conditions for the application, (iv) method by which the material(s) was processed into a device, (v) age of the material and its stability, (vi) defects, and (vii) macroscopic factors such as grain boundaries and cracking, which can strongly influence, for example, charge transport behavior in a solar cell device.

### Synthetic Realization Prediction

Given the significant efforts in computational materials discovery, including a large field in high-throughput computational screening, it is not unreasonable to ask why are there, by comparison, relatively few experimentally realized materials that originated from computational predictions? One reason is that a predicted material, albeit with very promising predicted properties, does not come with a “recipe”, or synthetic procedure.^28^ A materials synthesis procedure may require multiple stages, for example the synthesis of the precursors (such as the component organic molecules) and the synthesis reaction of those components into the desired material structure or topology, followed by processing of that material into the desired form for application, such as a multilayer device, thin film, or fiber. Each of these stages is typically time-consuming (often months) and expensive, and there is potential for failure at each step. For example in our work, even with porous organic materials designed by a synthetic chemist with the expectation of synthetic success, only 42% of the reactions were successful.^6^ For the synthesis of MOFs, while one could describe the synthesis as relatively simple, with one-pot mixing of the component materials, the material synthesis outcome and properties can vary depending on the conditions such as solvent choice and temperature, and the optimization of the phase diagram of the new material is far from trivial.^8^ Therefore, if computational predictions came with a “recipe” for how to make the target material, much like a retrosynthesis of an organic molecule, this would increase the number of successfully synthesized computationally predicted materials, and may be especially important for branching out into novel material space. However, how to achieve this universally is far from obvious.

### Need for Reliability and Trust in Computational Predictions

Strongly related to the previous points, there is the need for computational predictions to be trusted and expected to be reliable by the experimental researchers in the field. This is going to be particularly necessary to risk the synthesis of predictions in novel material space that are further from known materials, where an experimental researcher can automatically have a high degree of confidence in their synthesis. It is not unreasonable that there may be a high level of caution in the synthesis of predictions, in particular, given the many challenges in the accuracy of predictions that still face the field and the high financial and time cost in testing predictions. This hesitancy may go some way to explaining the relatively low number of experimental realizations of predictions—relatively few are attempted and those that are, are often very similar to previously reported materials. It is notable that many of the examples of experimental realizations of computationally predicted materials are carried out by experimental researchers who have a long history of working with computational researchers, or where the same researcher has carried out the prediction and then entered the lab to test it.^19,29^

### Limited Data Availability

The digitization of scientific literature, open access databases, and open-source algorithms have ushered in a new era of data-driven prediction and materials development. While there is a lot of excitement surrounding data-driven techniques in materials discovery, most successful implementations of these tools happen for data-rich problems, since data-driven techniques rely on statistics rather than existing physical equations governing material properties. Indeed, the success of these statistics-based analyses and prediction relies on possessing a reliable, diverse, and large data set. However, unless there is an existing, open-source, readily available database for a given class of materials, a data set must be constructed. Whether generating data via experiment or computation, this is an arduous and limiting task. Recently, there have been improvements to data set construction methods, including more efficient computation, online open source databases, data imputation techniques, and the implementation of text-mining tools to extract and process data in published articles and patents.^30,31^ Yet, there are still challenges and limitations associated with each of these methods. Text-mining the scientific literature has attracted a lot of attention;^32^ however, inconsistencies in chemical nomenclature, literature reporting, and synthesis summaries limit the number of articles from which data may be obtained.^33^ Issues further persist for experimental data mining because of potential inconsistencies in experiment reporting, and undisclosed environmental factors; for example, a commonly reported metric in syntheses is temperature; however, “room temperature” varies with geographic location. Moving forward there is a clear need for consistent data reporting schemes and common ontologies.

### The Challenge of Extrapolating to Unknown Materials Space

While there are numerous examples of computational materials development in the literature, they largely fall under *design* and *identification* schemes and stay within the region of previously explored local material space. Therefore, identified candidate materials are necessarily limited and advancements within this arena are concerned more with optimization of known materials than truly *novel* materials discovery. Discovery of truly novel structures, topologies, motifs—those that exist beyond known material space—within the typical computational framework is presently evasive and new approaches are necessary.

At the core, sampling unknown material space is concerned with generating motifs that have a greater dissimilarity to the initial material space. Yet, many of the existing data-driven techniques are robust under only small extrapolations to the initial training data set. For example, models trained to predict band gaps of fully inorganic perovskites would perform poorly for band gap prediction of organic semiconductors. Specific exploration techniques have been developed and are largely based on either global optimization methods or generative ML models. Yet, these are often limited to small (often drug-like) molecules, and problems surrounding chemical feasibility of generated systems persist, even before considering synthesizability. Moreover, owing to issues surrounding chemical feasibility, the present implementation of generative ML models favor known chemical space exploration over extrapolation to the unknown. Intuitively, this makes sense because it is challenging to model what we do not know; in this way, the efficient search process offered by global optimization approaches is advantageous, allowing us to manage the degree of extrapolation. Beyond the methods employed, purely computational endeavors in sampling unknown material space require robust synthesizability metrics and route prediction; the best way to accomplish this is through close, synergistic communication between experiment and theory directly.

### Challenges with Inverse Design

Inverse design, where one starts from a list of desired properties and works backward to the necessary material and material components, is typically considered the “holy grail” of materials discovery. It would be efficient compared to the need to screen thousands to millions of materials and, in an ideal world, would not be limited to existent material space, and thus able to identify optimal target materials from the entire novel material search space. However, Jansen and Schön argued that rational development of materials is a fallacy, because the thermodynamic viability of a material must be considered as part of the discovery process.^34^ They argue it is irrelevant if a hypothetical material has optimal properties if it is thermodynamically (or kinetically) unstable and as such could never be synthesized or operate in a given application. Instead, they put forward the necessity of screening processes that first assess the thermodynamic viability of hypothetical material candidates, only taking forward to property screening those materials that are energetically viable. Despite advances in hardware and computation software, computational stability assessment for hypothetical materials is extremely challenging,^35,36^ often relying on calculations of formation enthalpies with electronic structure methods. The energy landscape for a single material alone is often complex. Thus, from an inverse design perspective, selecting the optimal material candidate from a material space is highly computationally intensive and challenging.

## Progress in Computational Discovery

Despite the hurdles that the above barriers present to realizing the full potential of computation-led novel materials discovery, these are exciting, active areas of research within the community. Here, we highlight the state-of-the art in materials discovery initiatives that will allow computation to assist in the discovery of more diverse and novel materials.

### Advantages of Data-Driven Computational Discovery

There is enormous potential in the use of AI and data-driven techniques to overcome existent barriers to discovery through completely alternative approaches that elucidate subtle trends in high-dimensional data. This is being facilitated by open-source algorithms, for example, those for deep learning. Arguably, (materials) chemistry is behind other disciplines in the use of AI due to the lacking quantity and quality of data required for ML existing in machine-readable forms. This is particularly pertinent when many of the most interesting or useful instances in chemistry are “rare” and the exception to the rule. Low data quantity is often addressed by supplementing data sets with computational data, where generation has been facilitated by advancements in hardware and high-throughput computational methods and software.^37,38^ Notably, models trained on computational data exacerbate potential errors associated with the required assumptions, e.g. basis set superposition error within DFT.^39^ Although it could be possible to assume important relative relationships are sufficiently preserved, which enables these methods to guide experimental efforts. Further, we lack the “dark” data on experimental failures that algorithms need to include in their training. However, there have been enormous initiatives in the materials community to overcome this challenge, such as the Materials Project,^40^ Materials Genome Initiative,^41,42^ and Novel Materials Discovery (NOMAD),^43^ among others,^44−46^ and there is increasing awareness of the importance of open-source data and use of electronic laboratory notebooks. Below, we discuss the potential for data-driven approaches for specific tasks.

### Structure Prediction

Sufficiently accurate structure prediction is a key step in reliable prediction of material properties.^47^ Recent advancements in computational structure prediction methods have significantly improved the efficiency and accuracy of what still remains a costly procedure that requires specialists skills and typically weeks of calculations. Crystal structure prediction (CSP) is a powerful technique that predicts solid-state structure from knowledge only of the individual components. There are separate approaches for organic (molecular) systems and inorganic systems, and these fields have largely diverged.

**Figure 2:**
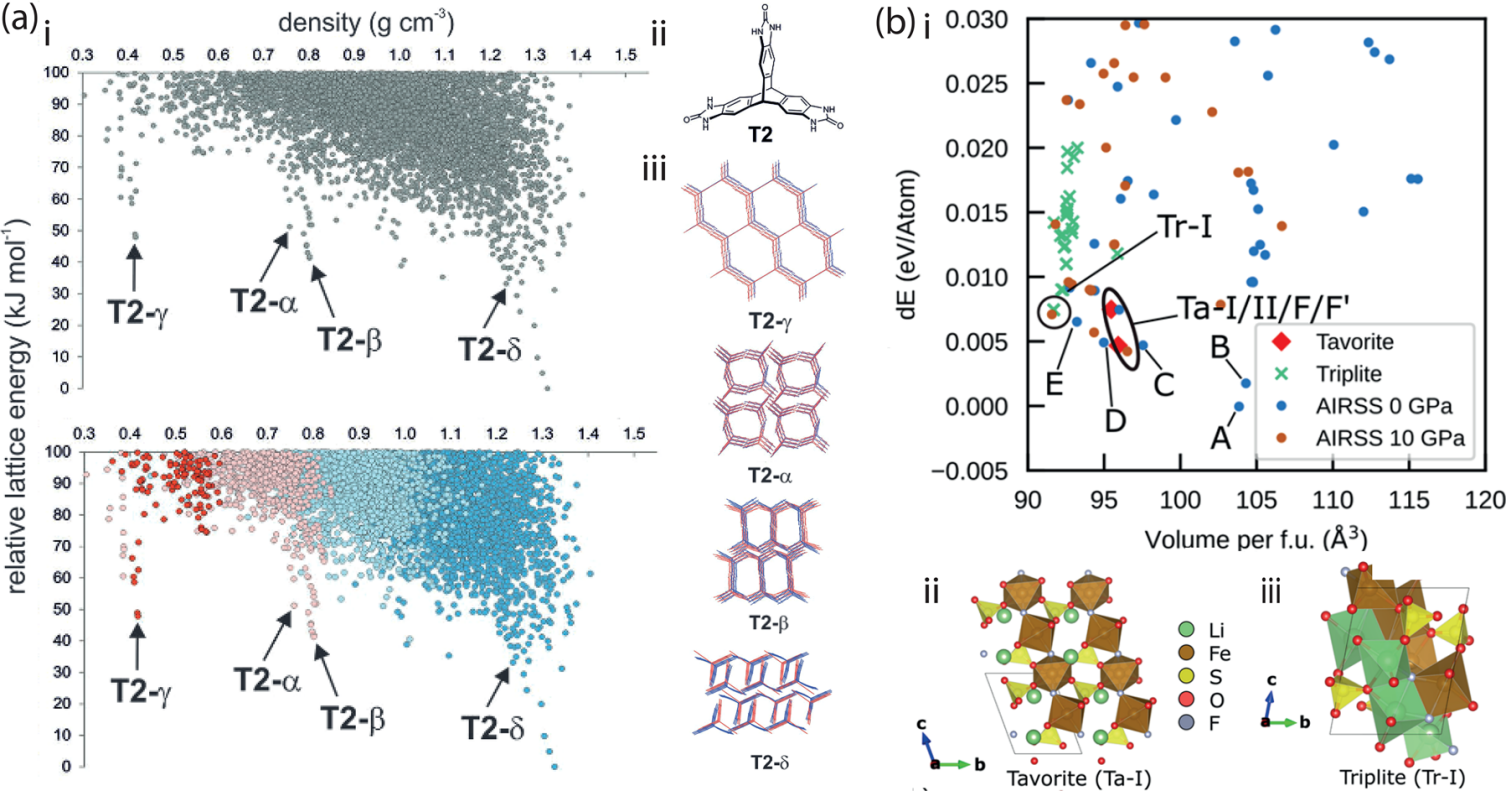
Structural prediction of novel (a) organic and (b) inorganic materials. (a) Crystal structure prediction to predict new porous materials. (i) The lattice energy landscape (top) and energy–structure–function map (bottom) of the molecular crystals of (ii) **T2**, highlighting the (iii) polymorphic behavior, leading to the experimental discovery of the highly porous **T2**-γ phase. Adapted with permission from ref (48). Copyright 2017 John Wiley & Sons, Inc. https://creativecommons.org/licenses/by/4.0/. (b) *Ab initio* Random Structure Searching (AIRSS) to explore complex cathode materials. The search (i) found both the experimentally known phases of LiFeSO_4_F (ii, iii) and identified new low energy polymorphs, some of which had favorable properties. Adapted from ref (20), *APL Materials***2021**, *9*, 121111, with the permission of AIP Publishing.

For organic molecules, the procedure generally involves sampling a large number of different crystal packings and then assessing their relative energies, under the assumption that experimentally observable structures will lie within a few kJ mol^–1^ of the global minimum. This allows for the creation of energy–structure–function relationships,^48^ guiding experimental discovery of targeted properties. For example, CSP was used to guide synthetic efforts to rigid molecular motifs likely to produce experimentally accessible and highly porous polymorphs from only knowledge of the molecular structure, Figure 2a.^19^ While not all the molecules studied in this work were new, the computational results highlighted that one could find a novel packing with novel properties, predicting the existence of an ultralow-density solid. This underlines the potential for computation to lead discovery beyond what can be achieved by experiment alone.

While structure prediction for inorganic materials benefits from stronger directional interactions compared to their organic counterparts, computationally led inorganic materials discovery still requires thorough sampling of possible phase space and accurate energetic ranking of low-energy phases.^49,50^ These methods have helped accelerate materials discovery, for example finding low-energy polymorphs of complex cathode materials with more favorable properties,^20^Figure 2b. Knowing that a particular polymorph is low-energy does not, however, mean it is trivial to access in the laboratory.

As evidenced by the blind structure prediction tests that are held every few years, what has been a “game-changer” for the accuracy of CSP is the use of electronic structure methods with high-quality descriptions of intermolecular interactions.^51,52^ However, using these methods to assess the energies of potentially hundreds of polymorphs per molecule is very computationally demanding, limiting the use of CSP in high-throughput screening workflows. It is even more computationally expensive to apply CSP to more complex systems, such as flexible molecules, multicomponent crystals, or systems where Z′ > 1 or large unit cells. However, the introduction of molecular complexity has the potential to discover more exotic phases in novel material space. The constant development of structure prediction techniques and compute power means more will be possible in the future, but it will remain challenging in the short term for CSP to explore large amounts of material space. In the meantime, we suggest simpler coarse-grained methods as a more accessible route to explore broader strokes of phase space, discovering regions for further exploration—both experimentally and with higher levels of theory—as well as developing design rules. Alternatively, ML force fields are a promising approach to improve the tractability of *ab initio* calculations via incorporation with classical force field methods.^53,54^ Indeed, ML force fields have been successfully applied, for example, in elucidating amorphous carbon structures^55^ and phases of sodium under pressure.^56^

Structure prediction is also used in other amorphous systems. For example, polymerization algorithms, via sequential bond forming and annealing steps, can reliably reproduce the structure of amorphous organic polymers in membranes.^57,58^ The amorphous structure prediction field is behind that of crystalline structure prediction and lacks the databases of thousands—millions of structures that would benefit identification and data-driven prediction, although work to build these is underway.^59^

The energetic viability of predicted structures is an important consideration. In a recent example, the synthesizability of crystalline materials represented by their atomic structure was predicted using deep learning.^60^ In this example, Davariashtiyani et al. present a synthesizability prediction model that is generalizable across materials classes assuming synthesis data availability. There have also been attempts to predict the thermodynamic limit of crystalline materials relative to a proposed “stability limit” based upon the energy of the amorphous material.^61^ Confident, reliable prediction of synthetically viable material structures through an energetic assessment of the possibilities will be a key step toward accessing novel material space, such as complex exotic phases or novel motifs. Increased reliability of structure prediction methods will likely have a direct and positive impact on the confidence of synthetic material chemists in attempted syntheses. The structure prediction field could best benefit novel materials discovery by decreasing the computational cost of energetic assessment of the hypothetical phases, such that the approach can be more routinely used in high-throughput materials discovery pipelines.

### Synthesis Route Prediction

Novel materials predicted by computation are only viable if they are experimentally realizable; thus, each predicted material would ideally be accompanied by a computationally predicted synthesis route. Yet, we are faced with an expansive reaction space to traverse, and different methods are necessary for different materials classes. For organic molecules, the primary component of organic materials, there has been extensive effort in computer-aided synthesis planning methods.^62,63^ These retrosynthetic planning methods are either template-based, where novel synthetic routes are predicted from existing databases of reactions,^64^ or template-free, which do not rely on existing reaction templates and rely on data-driven techniques.^65,66^ The latter approach has obvious advantages for the prediction of novel systems, by not relying on prediction based upon existing knowledge. While these methods are still under development to reach the sophistication of an experienced human organic chemist, the equivalent planners for novel material synthesis are needed.

**Figure 3:**
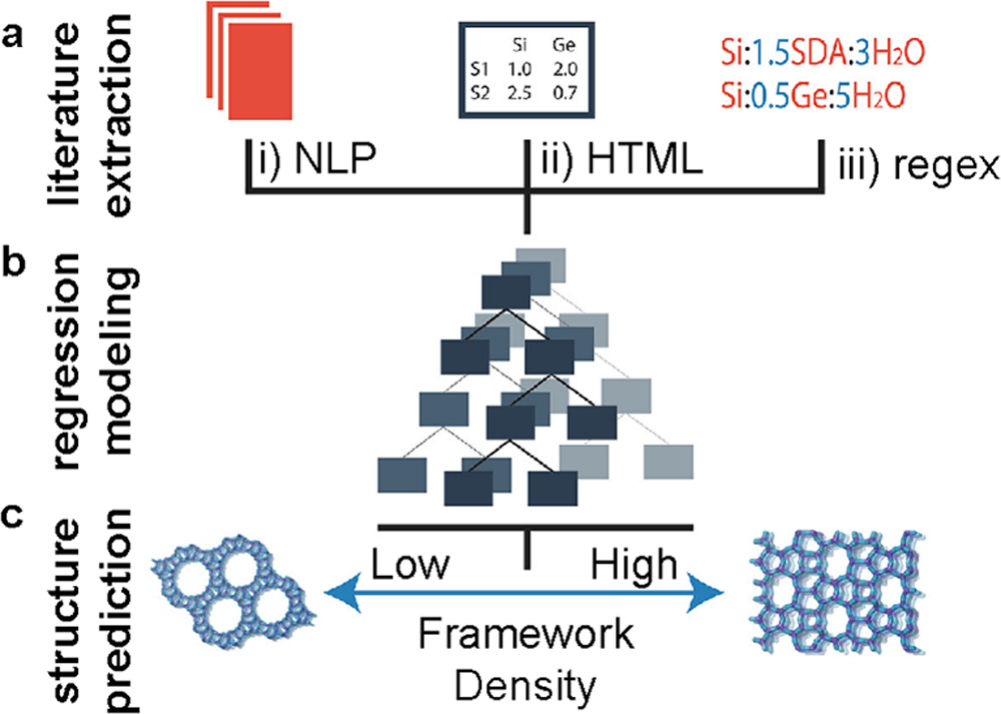
Representation of zeolite data engineering: (a) extraction and combination of synthesis data to (b) model and (c) predict framework density based upon synthesis route. Several tools were used for literature extraction, including (i) NLP, (ii) HTML table parsing, and (iii) compositional ratios from Regular Expressions (regex). Adapted with permission from ref (67). Copyright 2019 American Chemical Society.

Materials synthesis is typically far more complex than organic molecular synthesis, necessitating consideration of the precursor synthesis and conditions, in addition to the synthesis of the material itself, and there are limited examples in the literature for solid-state synthetic reaction route prediction. The accuracy of data-driven synthetic route prediction depends on the available synthetic data for the material of interest, and experimental data availability is highly field-dependent. In fields where there is a lack of existing synthetic information, databases of reaction templates can be constructed. One way to build these databases is via text-mining of the scientific literature and patents, which often relies on natural language processing (NLP) algorithms.^30,31^ NLP methods were recently used to extract syntheses and macroscopic field trends from published articles concerning zeolites.^67^ Here, Jensen et al. were able to successfully use the extracted synthesis data to elucidate the high-dimensional relationship between synthesis and product topology via a framework density prediction model. This work demonstrates a step toward novel zeolite topology discovery via synthesis data analysis and possesses a workflow featuring literature extraction, regression modeling, and structure prediction, Figure 3. Unfortunately, published synthetic methods are not consistent and highly variable. Recently, there has been a push toward establishing ontologies for reporting material synthesis (and properties) that would increase the machine readability of methods sections.^33^

Beyond route prediction, experimental conditions such as temperature, pressure, and solvent directly impact yield and product identity. Thus, conditions are an indispensable aspect of route prediction and there have been successful demonstrations of data-driven optimization of experimental conditions for given reactions,^68^ including target reaction properties, such as reaction yield,^69,70^ temperature and chemical context models,^71^ and solvent selection.^72,73^ In one material-specific example, data-driven techniques were used to optimize reaction conditions for the MOF HKUST-1.^8^ Here, Moosavi et al. specifically incorporated “failed” experimental data to decrease bias^74^ and used genetic algorithms and ML to optimize synthesis strategies in a generalizable workflow that was transferable between different inorganic components.

The synthetic routes and proposed optimal experimental conditions identified via these methods are only as good as the existing data allow. Verification of the predictions is necessary for improved performance—this is especially true for predicting synthetic routes and conditions for novel materials, and even more challenging. Improvement of these methods is enabled by a close, synergistic relationship with experiment. Optimization of synthetic route prediction algorithms could be achieved in an active learning, closed-loop, experimental-theoretical process, made efficient by implementation of high-throughput, autonomous robotic platforms; this is discussed further in the following sections.

### Property prediction

Properties associated with a given material structure may be obtained either from direct simulation or via data-driven methods. For the majority of the properties of inorganic materials, we rely on comparatively computationally expensive electronic structure calculations. Advancements in computational hardware, software, and methods are enabling electronic structure calculations to be carried out on both larger systems and on larger scales—increases in system size and number have the potential to continue to dramatically expand the size of the material space being explored.

However, the increasing ability to use data-driven approaches to predict the properties of novel materials is arguably the biggest “game changer” in the area of property prediction. Large data sets of material structures and their properties can be used to train supervised ML models, often via GPUs (graphics processing units), to predict those properties. Replacing direct simulation with an ML model accelerates prediction by orders of magnitude, translating to orders of magnitude larger material space being explorable, greatly increasing the potential discovery of novel materials. While data-driven approaches will allow effectively complete interpolation of local materials space, there remains danger in extrapolating the model far from where it has been trained. But, careful application of the models, for instance in selecting the next materials to directly test via either experiment or direct simulation during the exploration process, can reduce this danger.

The expanded use of data-driven methods is founded upon the existence of reliable data sets, and these are becoming increasingly available in the materials field resulting from both the drive to increase open-source (experimental and computational) data and the increase in computer power and thus the ability to conduct large scale generation of computational data for training data sets. Establishment of protocols for the reporting of materials properties is also important to ensure machine-readable formats.^75^ For example, the adsorption file format is a new standardized file format that is both human- and machine-readable and was recently proposed for reporting adsorption data in porous materials.^76^ Additional recommendations for the standardization and formatting of computational data have also been suggested;^77^ this object-oriented structure would promote the utility of community contributions. Indeed, standardized reporting formats and data structures are imperative to the accelerated discovery of novel compounds because they eliminate the necessary, but time-intensive, data collation step in computational materials discovery workflows.

**Figure 4:**
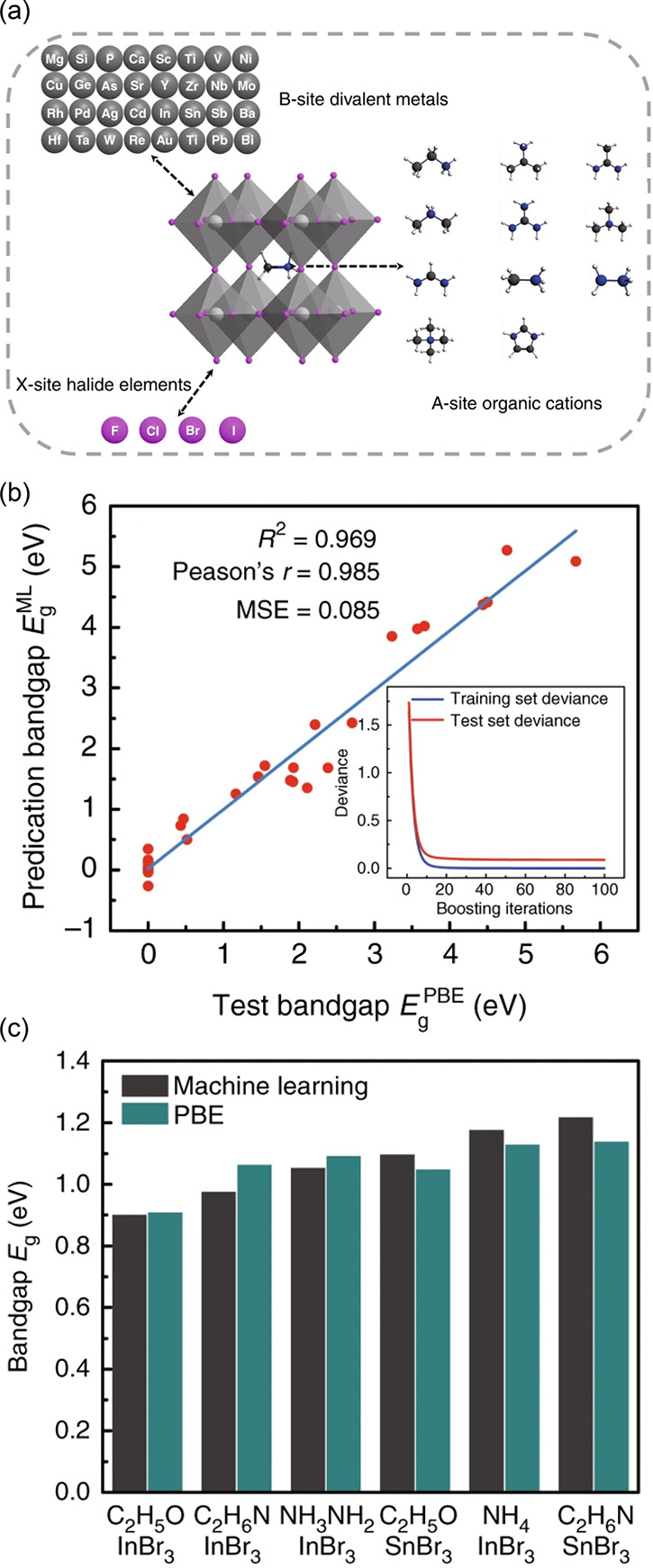
(a) Chemical space: A data set of HOIPs was combinatorially generated from libraries of A-site cations, B-site metals and X-site halides. (b) Model training: The performance in predicting bandgaps. The subplot is the convergence of model accuracy for five cross-validation split of the data. (c) Validation and implementation: Comparison between ML-predicted and DFT-calculated results of six selected HOIPs. Reprinted with permission from ref (78). Copyright 2018 Springer Nature. https://creativecommons.org/licenses/by/4.0/.

Property prediction facilitated by data-driven methods undoubtedly has the potential to significantly accelerate the discovery of novel materials featuring desired properties. One example is the use of data-driven methods to accelerate the discovery of hybrid organic–inorganic perovskites (HOIPs).^78^ Here, Lu et al. trained an ML model to predict the band gaps of known perovskites and later apply the model to unexplored hybrid perovskites, identifying six stable lead-free perovskites, Figure 4. The property prediction model in this case circumvented additional, costly band gap calculations that would otherwise be necessary to assess the performance of the unexplored materials. However, this is still an example of materials design, as opposed to novel materials discovery, which necessitates sampling outside of known material space.

### Sampling in Unknown Material Space

At their essence, novel materials discovery initiatives require improved methods to sample unknown material space. If one does not know what this material space even *looks like*, then it is hard to strategize the enumeration and exploration of that space. Present examples of exploration in unknown chemical space in the literature are sparse and mostly feature molecular systems, owing to their simple construction and synthetic routes. Indeed, extrapolation to unknown chemical space gets more complex as you move from molecules to materials because of the increased degrees of freedom. In one recent example in the materials literature, a new multiobjective, regression-based screening process to identify high-performing material candidates that exist in sparsely populated material space was presented.^79^ The basis of this method is the novel loss functions that enable the model to favor compounds possessing greater chemical dissimilarity to the original data set. While this is a screening approach, modifications to this workflow, wherein generative models are introduced, may show promise in the discovery of novel materials. Here, we discuss developments that can allow novel material space to be accessed through global optimization strategies and generative ML models.

(i) **Global optimization strategies.** The utility of global optimization strategies lies in their efficient exploration of material space and ability to perform multivariable optimization. Ideally, we would want to implement methods to allow effective exploration of *novel* materials space. Optimization approaches include basin hopping methods, quasi-random search methods, and evolutionary algorithms (EAs). Specifically, in quasi-random structure search and basin hopping approaches, existing computational tools can be augmented to access novel structures. For example, quasi-random structure search methods have been employed to discover novel crystal phases,^80^ in an intuitive expansion of CSP. Alternatively, within an application of the basin hopping method, initial velocities for molecular dynamics calculations were tuned to encourage more diverse paths.^81^

**Figure 5:**
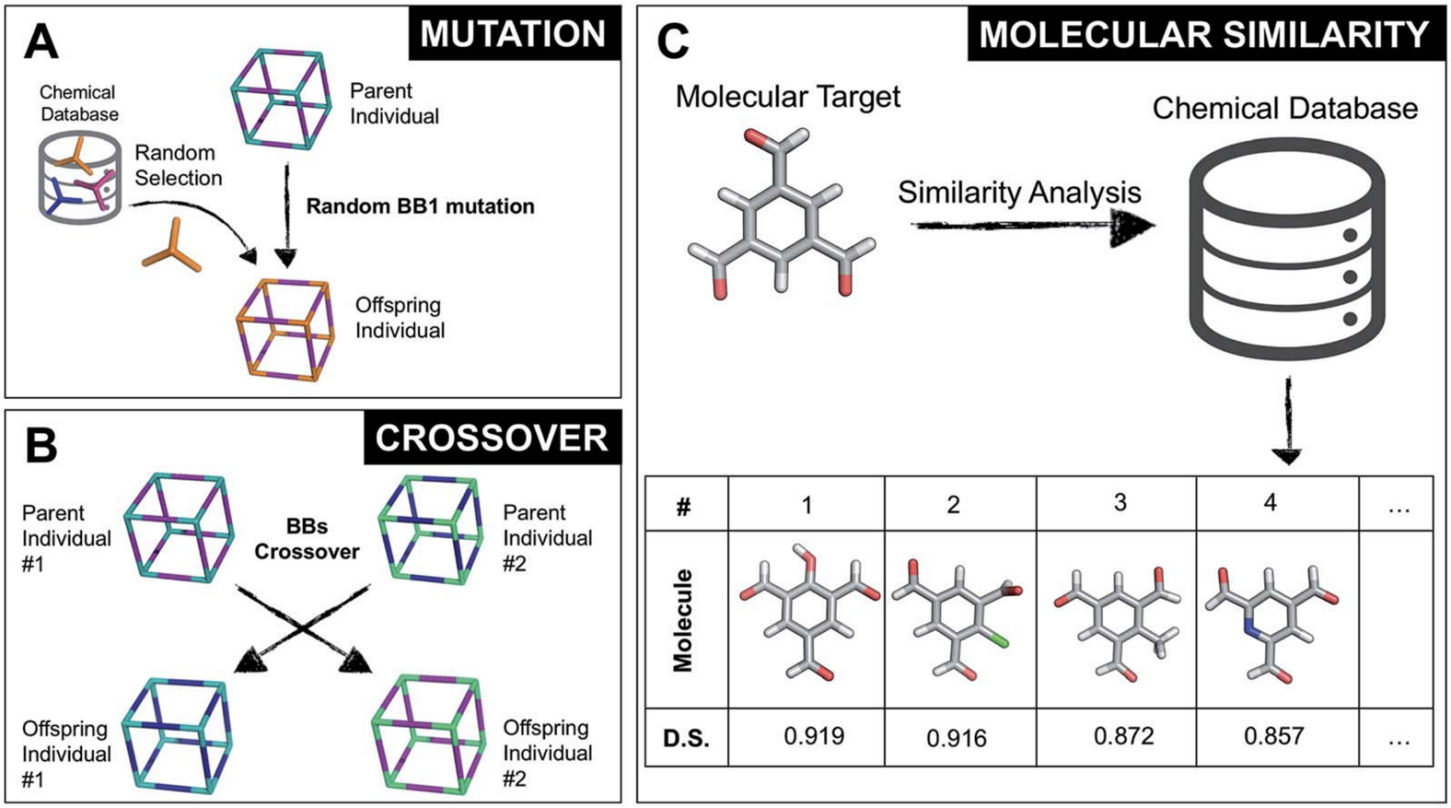
Example implementation of an EA. (a) Mutation operation for a two-component cage molecule where building block one (BB1) is randomly replaced. (b) Crossover event featuring two parents and their offspring. (c) Validation via molecular similarity to assess the distance between the molecular target and the chemical database to ensure most chemically feasible molecules are targeted. Reproduced from ref (82) with permission from the Royal Society of Chemistry. Copyright 2018 Royal Society of Chemistry. https://creativecommons.org/licenses/by/3.0/.

**Figure 6:**
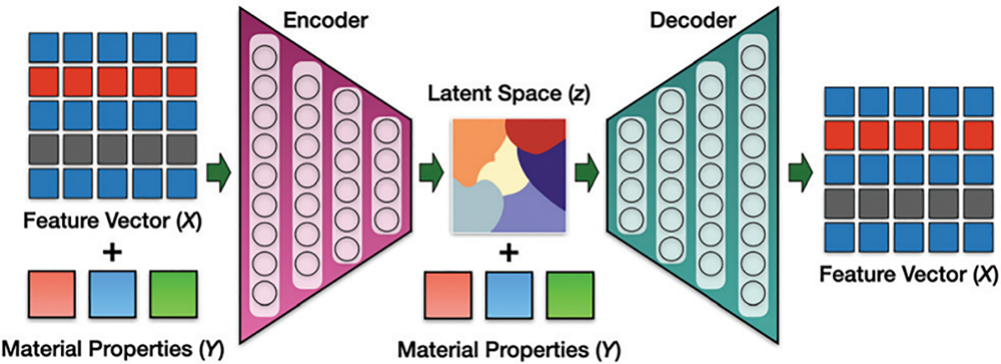
Schematic showing the variational autoencoder employed by Pathak et al.^87^ Materials are represented as a feature vector and encoded to the latent space via a neural network. New materials are generated by sampling the latent space via the decoder (neural network). Reproduced from ref (87) with permission from the Royal Society of Chemistry. Copyright 2020 Royal Society of Chemistry.

EAs simulate biological evolution events by implementing mutation, crossover, and selection in the model^82−84^ and, depending on the implementation, have great potential to explore novel material space; Figure 5 outlines an implementation of EA for porous organic cage design. However, if the EA uses a library based on known fragments and no additional steps where these potentially get modified, then there is no potential for exploring novel material space. Augmentation of these optimization methods are necessary to realize their full utility in novel materials discovery. For example, coupling EAs with generative ML models for fragment library generation would enable moving to unknown material space.

(ii) **Generative models.** Increasing data set size and availability have propelled the use of data-driven techniques in sampling unknown chemical space via generative ML algorithms. In general, these models work by decomposing materials and associated properties to a continuous vector representation. This latent space, learned from the training data, is then sampled to generate new chemical systems, or you can train models to generate data that are similar to the training data. While powerful, these models are often prone to generating invalid chemical motifs and are limited by the diversity of the initial training data set. Types of generative models include variational autoencoders (VAEs) and generative adversarial networks (GANs). VAEs encode materials (via a neural network) to a latent space, where properties are represented as probabilistic distributions. The latent space is sampled and processed by a decoder (neural network) to yield novel candidates and compositions, Figure 6. Conversely, GANs identify subtle trends and patterns in training data and exploit these learned patterns to generate artificial data that is similar to the training data set; this is accomplished by concurrently training two neural networks, (i) a discriminator, which determines the validity of the generated data, and (ii) a generator, which generates new data points from noise. Beyond VAEs and GANs, we have previously generated new molecules with target optoelectronic properties using a recurrent neural network, trained to produce SMILES strings of organic molecules, in conjunction with transfer learning.^85^ While generative methods have seen some success in the literature,^86,87^ utility is limited by their ability to generate chemically feasible *and* unique (novel) structures.

As with global optimization methods, generative models are trained on existing materials, which inherently restricts their ability to sample unknown chemical space. However, some techniques have been presented to avoid overfitting, which promotes novelty in the ensuing predictions.^88,89^ For example, enhanced novelty was achieved for small molecules via a recurrent neural network-based autoencoder trained to reconstruct molecular representations;^89^ Bilsland et al. further demonstrate that novelty is improved by purposefully incorporating invalid and known, undesirable SMILES into the model. In an alternative approach to mitigate bias by including “negative” examples, a VAE was recently used to generate a large, diverse, synthetic data set of balanced chemical reactions for subsequent ML training models.^90^ Using this technique, 7,000,000 novel reactions were generated from a model trained on only 7,000; indeed, the generated reactions feature a more diverse set of molecules than was present in the training set. It is important to note that within the context of generative models, “novelty” refers to whether a generated structure is present in the training data set—not whether the generated structure is truly known. Thus, to assess the true “novelty” of a candidate with respect to known chemical space, similarity metrics comparing candidates to material systems outside the training set must be implemented. Indeed, progress in model development and materials representations will fuel progress across materials discovery. In a recent example, a GAN was trained on full material representations consisting of atomic and energetic information on known zeolites and used to generate crystalline porous materials. Through additional parameters, users are able to tune the target property of the generated structure within a desired range.^91^ Yet, the GAN presented in this study is still problematic with respect to “novelty” because it is trained specifically to generate tensors similar to those it was trained on. This is novelty within the zeolite material class in its own right (constrained novelty).

While model and representation selection is a problem-specific task, methods may be linked with material representation. For example, reticular materials, which are constructed from molecular building blocks, benefit from molecular discovery advancements since they may be treated as fragments in EAs^82,92,93^ or readily encoded to a latent space.^94^ A VAE was recently used to generate reticular materials trained on supramolecular species;^94^ here, notably, the designed material search space encoded to the latent space was constructed to exclude new topologies as a way to promote experimental realizability. With necessary advancements to structure and energetic stability prediction, as well as synthesis route prediction, these precautions may not be necessary and exploration beyond known material space may be promoted. Table S1 summarizes the presented examples of materials discovery.

While an integral part of theory-driven computational materials discovery, it is not enough to predict novel motifs. They must also be realizable. There are three major metrics that must be used to assess the viability of novel structures obtained from sampling unknown chemical space: (i) structure feasibility, the structure must reasonably follow known chemical rules, (ii) formation energy, a valuable indicator of material stability, and (iii) realizability, we must be able to experimentally synthesize the predicted material.^95^ The recognized need to quantify these metrics has resulted in a series of published, predictive models, including MatLearn,^95^ a web-based predictive formation energy model specifically designed for utility by noncomputationalists to guide discovery initiatives toward regions of chemical space exhibiting higher degrees of thermodynamic accessibility. This approach is specific to inorganic compounds, yet we can envision a logical extension to other material classes. The necessary predictive models for assessing novel structure validity motivate close communication between experiment and theory to improve accuracy of realizability metrics.

### Close Synergy of Experiment and Theory

These are exciting times both for experimental materials discovery, with the increasing usability and decreasing cost of automation platforms and robotics enabling larger scale screening, and for computation, with data-driven approaches accelerating property prediction and opening new exploration avenues. We would suggest that these methods can be used most powerfully when combined rather than in isolation.^29^ While issues with the experimental realization of computational predictions remain, one might also imagine approaches attempting to explore as wide a material space as possible on an automation platform, and only when a preliminary “hit” of a material is found experimentally will the material’s properties be predicted. Thereafter, only the most promising materials will be fully characterized in the laboratory. An increasing number of experimental chemists and materials scientists have a high degree of computational literacy, including coding, which is often necessary for interfacing with automated platforms, as well as processing and analyzing large quantities of data autonomously. This allows smooth transitions between different tasks for the close synergy of experiment and theory, in addition to building trust on both sides–a key point highlighted earlier in this perspective.

While computation can guide the search space for high-throughput experimental screening,^96^ screening can also generate larger data sets for ML, where there can be more certainty on the consistency of the synthesis and measurement procedure. These data will be invaluable for both property prediction and synthesis route and condition prediction. It is not trivial to automate the majority of material synthesis or characterization tasks. Indeed, for many tasks it is implausible that this will ever be possible, specifically cases where large-scale, expensive characterization hardware is required. Automation platforms are typically setup for a specific project/workflow and are arguably best tasked to optimize a known phase space. For example, which combination of components optimizes a property, rather than be able to truly explore material space. The latter is an inherent limitation based on availability and automated provision of chemical starting materials to the robotic platform, in addition to the work required to setup, test, and validate the platform. While this process has recently been fully realized for inorganic supramolecular systems,^97^ photocatalyst mixtures,^98^ and organic synthesis,^99^ it is not necessarily feasible for all other systems owing to more complex synthetic procedures, delicate experimental steps, etc. Ultimately, this means that with high-throughput experimentation alone, it is unlikely that novel materials architectures will be discovered. Next, we discuss methods to strengthen communication between experiment and theory: (i) reinforcement learning techniques and (ii) closed-loop discovery workflows.

(i) **Reinforcement learning.** The necessary synergy of experiment and theory may be best facilitated by data-driven tools, such as reinforcement learning techniques, which benefit from a reward-based feedback loop with a ML model directing the next experimental action/selection. These models, which do not require large amounts of initial data, are ultimately concerned with what to do next considering the current knowledge. The ensuing cycle rewards positive behaviors and punishes negative behaviors to achieve an optimal solution. Within the context of materials discovery, decisions leading to experimental “hits” are positive behaviors, while experimental “failures” are negative behaviors. This type of exploratory tool is especially useful to explore large materials spaces^93,100^ or optimize chemical reactions,^101^ demonstrating its utility and potential integration at multiple levels of the discovery process. Specifically within the context of novel materials discovery, these tools have been used to identify optimal defect configurations in 2D materials,^102^ as well as phase diagram construction.^103^

**Figure 7:**
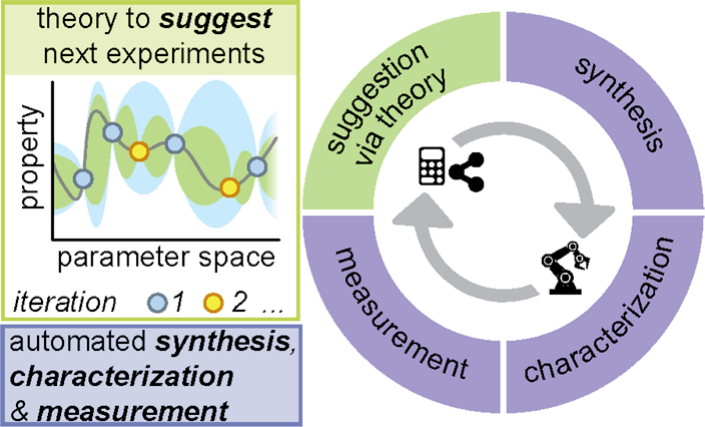
A closed-loop materials discovery workflow consists of four major parts, where ML-driven suggestions are fed into an automated experimental platform for subsequent synthesis, characterization, and measurement.

Reinforcement learning is positioned to strengthen the synergistic relationship between experiment and theory that is necessary for novel materials discovery. The development of robust, predictive computational models relies on theory’s ability to adequately replicate/represent reality—this is inherently a feedback loop problem that would benefit from constant rewards-based communication with experiment. Thus, reinforcement learning often is the backbone of closed-loop, experiment-theory discovery processes.

(ii) **Closed-loop discovery.** Closed-loop discovery is a seamless example of the close integration of experiment and theory. In such discovery processes, an initial set of experiments is tested on an automated platform, followed by automated analysis of the outcome (for our purposes the materials’ properties) and subsequent use of an optimization algorithm to select the next set of experiments, Figure 7, with the goal of improving material performance. This process is then iterated until the convergence criteria are met in a workflow suited to active learning algorithms. Active learning algorithms identify the next set of experiments based on regions of space that are poorly understood; the model is improved in an iterative process. Recently, a form of active learning autonomously directed real-time X-ray diffraction measurement experiments toward discovering novel phase-change memory materials.^104^ Here, promising next candidates to measure were selected from a materials database featuring both experimental and computational data using a physics-informed active learning model. As presented, this is an optimization problem. However, with intuitive expansions to this workflow, such as autonomous synthesis and generative models, materials discovery may be realized.

The selection of algorithm and implementation in closed-loop discovery will be key to exploring more novel material space. First, the initial set of experiments can be selected by an algorithm so as to cover the most chemically diverse materials within the possible options. Then, the algorithm that selects the next set of experiments to conduct generally has two tasks, to (i) build a model that predicts the property of a material and (ii) seek to optimize the property of new materials tested. In particular, Bayesian optimization is frequently used. This technique is a global optimization strategy promoting directed search to optimize an unknown function, in our case a material property. This is accomplished via a surrogate model that predicts a materials property as a function of its position in material space. Candidate selection is directed by a function that seeks a balance of *exploitation*, improving the material performance, and *exploration*, ensuring effective global optimization is achieved rather than just optimizing the local search space.^105^ By modifying the hyperparameters, the degree of exploitation vs exploration can be shifted so as to favor exploration, which has a greater potential to return novel structures.

A recent example of closed-loop discovery via an automated robotic platform comes from Burger et al., where they developed a mobile robot to test photocatalysts for hydrogen production from water.^98^ The robot performed 688 experiments over 8 days to explore a ten-variable search space, achieving six times more active formulations than those tested in the first step through via Bayesian optimization. This is extremely impressive, although it initially takes a significant amount of time to set up the mobile chemist to perform the necessary tasks; it is adaptable to other types of tasks and would be more adaptable than a single automated platform. The difficulties in using these approaches for global materials space exploration, mentioned above, remain.

## Conclusions and Outlook

It is a challenge to truly achieve *novel* materials discovery rather than exploring local regions of material space. Yet, this does not detract from the fact that both materials design and identification approaches have significantly accelerated theory-driven materials development by identifying promising candidates. Novel materials discovery is an understandably challenging task; this is further compounded by the fact that it is unclear whether known laws persist in unknown materials space—for example, consider hydrocarbons with extraordinarily long C–C bonds. Conventional chemical rules dictate a C–C bond is 1.54 , yet compounds exhibiting increasingly longer C–C bonds have been presented,^106,107^ with the record being 1.93 .^108^ Since theory is derived from our existing understanding of chemical behavior, it is incredibly challenging to computationally explore outside beyond this and still maintain some degree of chemical feasibility. Should the record-holding C–C bond compound have been computationally predicted first, it would likely have been met by skepticism. Perhaps through a close, synergistic relationship between experiment and theory, we may eventually be able to construct models that allow us to predict truly novel materials of this nature with confidence that they can be experimentally realized.

In order to reach unknown materials space, we must start with what is known, and while quantifying the size of a chemical space is useful, there are far more materials thought to be stable than atoms in the solar system. Presently, we have the tools to enumerate known chemical space, as we know it—and we are just beginning to see the emergence of tools to efficiently search this vast space. These developments, including data-driven techniques, high-throughput computational and experimental techniques, and robotic systems, could significantly decrease the materials discovery time. While the advances made in these areas are of significant note, there is still room for optimization and improvement of both theoretical and experimental methods and protocols. Indeed, the dream of a generalized, fully automated materials synthesis robotic platform has yet to be realized, in part because of the complexity of materials synthetic protocols; more novel materials may require more complex synthesis routes that are not presently available at automated platform scale.

Ultimately, efforts in materials discovery initiatives must be focused on improving several areas: (i) high-throughput experimental materials synthesis platforms, (ii) increasing the efficiency of solid-state material property calculation, (iii) synthetic accessibility scores and synthetic route predictions, and (iv) improved methods for exploring beyond known chemical space. Accomplishing many of these tasks will require dedicated teamwork between experimental and computational scientists. This relies heavily on trust between the two communities and is necessary to develop robust *predictive* computational models, moving the field into a new era of novel materials discovery.

## Supporting Information Available

Summary of the highlighted examples in materials discovery. (PDF)
