# Supplementary material for: Into the Unknown: How Computation Can Help Explore Uncharted Material Space
Source: J Am Chem Soc. 2022 Oct 7;144(41):18730–43. doi: 10.1021/jacs.2c06833 (PMC9585593; doi:10.1021/jacs.2c06833)
Supplement: Supplementary file 1 — ja2c06833_si_001.pdf [file ja2c06833_si_001.pdf]

Supporting Information: Into the Unknown: How Computation Can Help Explore Uncharted Material Space

Austin M. Mroz,† Victor Posligua,†,‡ Andrew Tarzia,†,‡ Emma H. Wolpert,†,‡ and Kim E. Jelfs*,†

†*Department of Chemistry, Molecular Sciences Research Hub, Imperial College London, White City Campus, Wood Lane, London, W12 0BZ, UK*

‡*These authors contributed equally*

E-mail: k.jelfs@imperial.ac.uk

Table S1: Summary of model type, chemical representation, materials types and target properties for selected examples of materials discovery.

Model	Representation	Materials Class	Target Property	Ref.
GANs	3D potential energy grid	zeolites	CH_4 heat of adsorption	[S1]
Conditional VAE	feature vector of elemental composition	inorganic materials	enthalpy of formation	[S2]
VAE	SMILES (reticular building blocks)	MOFs	CO_2 separation	[S3]
RNN/ANN	SMILES	Small molecules	Novelty	[S4,S5]

References

(S1) Kim, B.; Lee, S.; Kim, J. Inverse design of porous materials using artificial neural networks. *Science Advances* **2020**, *6*, eaax9324, doi: 10.1126/sciadv.aax9324.

(S2) Pathak, Y.; Juneja, K. S.; Varma, G.; Ehara, M.; Priyakumar, U. D. Deep learning enabled inorganic material generator. *Physical Chemistry Chemical Physics* **2020**, *22*, 26935–26943.

(S3) Yao, Z.; Sánchez-Lengeling, B.; Bobbitt, N. S.; Bucior, B. J.; Kumar, S. G. H.; Collins, S. P.; Burns, T.; Woo, T. K.; Farha, O. K.; Snurr, R. Q.; Aspuru-Guzik, A. Inverse design of nanoporous crystalline reticular materials with deep generative models. *Nature Machine Intelligence* **2021**, *3*, 76–86.

(S4) van Deursen, R.; Ertl, P.; Tetko, I. V.; Godin, G. GEN: highly efficient SMILES explorer using autodidactic generative examination networks. *Journal of Cheminformatics* **2020**, *12*, 22.

(S5) Bilsland, A. E.; McAulay, K.; West, R.; Pugliese, A.; Bower, J. Automated Generation of Novel Fragments Using Screening Data, a Dual SMILES Autoencoder, Transfer Learning and Syntax Correction. *Journal of Chemical Information and Modeling* **2021**, *61*, 2547–2559, doi: 10.1021/acs.jcim.0c01226.
